# Blood-Brain Barrier Impairment in an Animal Model of MPS III B

**DOI:** 10.1371/journal.pone.0016601

**Published:** 2011-03-07

**Authors:** Svitlana Garbuzova-Davis, Michael K. Louis, Edward M. Haller, Hiranya M. Derasari, Ashley E. Rawls, Paul R. Sanberg

**Affiliations:** 1 College of Medicine, Center of Excellence for Aging & Brain Repair, University of South Florida, Tampa, Florida, United States of America; 2 Department of Neurosurgery and Brain Repair, College of Medicine, University of South Florida, Tampa, Florida, United States of America; 3 Department of Molecular Pharmacology and Physiology, College of Medicine, University of South Florida, Tampa, Florida, United States of America; 4 Department of Pathology and Cell Biology, College of Medicine, University of South Florida, Tampa, Florida, United States of America; 5 Electron Microscopy Core, Department of Integrative Biology, University of South Florida, Tampa, Florida, United States of America; 6 Department of Psychiatry, College of Medicine, University of South Florida, Tampa, Florida, United States of America; French National Centre for Scientific Research, France

## Abstract

**Background:**

Sanfilippo syndrome type B (MPS III B) is caused by a deficiency of α-N-acetylglucosaminidase enzyme, leading to accumulation of heparan sulfate within lysosomes and eventual progressive cerebral and systemic multiple organ abnormalities. However, little is known about the competence of the blood-brain barrier (BBB) in MPS III B. BBB dysfunction in this devastating disorder could contribute to neuropathological disease manifestations.

**Methodology/Principal Findings:**

In the present study, we investigated structural (electron microscope) and functional (vascular leakage) integrity of the BBB in a mouse model of MPS III B at different stages of disease, focusing on brain structures known to experience neuropathological changes. Major findings of our study were: (1) endothelial cell damage in capillary ultrastructure, compromising the BBB and resulting in vascular leakage, (2) formation of numerous large vacuoles in endothelial cells and perivascular cells (pericytes and perivascular macrophages) in the large majority of vessels, (3) edematous space around microvessels, (4) microaneurysm adjacent to a ruptured endothelium, (6) Evans Blue and albumin microvascular leakage in various brain structures, (7) GM3 ganglioside accumulation in endothelium of the brain microvasculature.

**Conclusions/Significance:**

These new findings of BBB structural and function impairment in MPS III B mice even at early disease stage may have implications for disease pathogenesis and should be considered in current and future development of treatments for MPS III B.

## Introduction

Mucopolysaccharidoses (MPS) are inherited lysosomal storage disorders caused by malfunctioning of particular lysosomal enzymes. The resulting accumulation of undegraded glycosaminoglycans (GAGs) within lysosomes leads to progressive cellular damage affecting normal organ and system function. Sanfilippo syndrome type B (MPS III B) is one of four MPS III (Sanfilippo syndrome) subtypes and is characterized by a deficiency of α-N-acetylglucosaminidase (*Naglu*), the enzyme responsible for degradation of heparan sulfate (HS). Accumulation of HS in lysosomes leads to progressive cerebral and systemic multiple organ abnormalities. Clinical symptoms such as hyperactivity and aggressive behavior appear between two and six years of age and are followed by hearing or vision defects, pathological skeletal changes, and mental retardation [1], [2]. MPS III patients experience pathological changes in the brain characterized by varying degrees of cortical atrophy and ventricular enlargement [3], [4]. In some patients, callosal atrophy and cerebellar changes were noted along with morphological abnormalities in Purkinje cells [5]. Death usually occurs between 11 and 20 years of age. Treatment of patients with MPS III B is mainly supportive.

The disorder is caused by mutations in the *Naglu* gene located on chromosome 17q21, a gene encoding a lysosomal enzyme needed for degradation of heparan sulfate [6]. A knockout mouse model of MPS III B, presenting biochemical abnormalities similar to the human disease, has been created by disrupting this gene [7]. The *Naglu* deficient mice show progressive deterioration of motor, vision, and hearing functions [8]. Histopathological changes such as vacuolization, observed in multiple mouse organ systems as early as the first month of age, become more prominent over time. Initial symptoms present at about 6 months and mice survive until 8 to 12 months of age [7]. Neurons are affected in various parts of the brain such as the olfactory bulb, cortex, thalamus, amygdala, midbrain, pons, medulla, and cerebellum, leading to neuropathological alterations. Accumulation of gangliosides GM2 and GM3, secondary storage products, and formation of large cytoplasmic inclusions have been reported in various brain cell types [7], [9], [10]. Additionally, progressive increases in reactive astrocytes in the cerebral cortex and hippocampus of 3 month and 6 month old *Naglu* mice [11], activated microglia [12], and the presence of pro-inflammatory cytokine IFN-γ in the mouse cerebral cortex at 1 month of age [12] indicate inflammatory processes in the brains of mice modeling MPS III B.

Thus, MPS III B presents a complexity of pathological factors. However, little is known about the condition of the blood-brain barrier (BBB) in MPS III B. BBB dysfunction in this disorder could contribute to neuropathological disease manifestations by accelerating cell death. The primary function of the BBB is controlling cerebral homeostasis by selective transport of molecules and cells from the systemic compartment [13]–[15]. The BBB is composed of the unique structural elements of the microvasculature – endothelial cells of brain capillaries, epithelial cells of the choroids plexus, astrocyte end-feet, and pericytes. Endothelial cells are connected via adherens and tight junctions, forming a diffusion barrier. Functional integrity of all BBB elements is critical for protection of the CNS from harmful blood substances. Substances with molecular weights greater than ∼400 Da are too large to cross the BBB by free diffusion. Compromised BBB integrity has been demonstrated in some lysosomal storage diseases (reviewed in [16]). Extravasation of Evans Blue and endogenous IgG was found in mouse models of GM2 (Sandhoff disease) and GM1 gangliosidoses [17]. Interestingly, IgG leaky vessels were more pronounced in the stratum radiatum of the hippocampus, medial thalamus, and the inferior colliculus in Sandhoff mice at late stage of disease. In MPS II and MPS III B patients, dilatation of the perivascular space around the brain capillaries was noted in the cerebral white matter [18]. However, no data exist about structural and functional BBB state in MPS III B, specifically in various brain structures and at different disease stages. Determining BBB condition in MPS III B is crucial to understanding additional mechanisms of disease pathogenesis and to developing pharmacological and/or cellular treatments.

The aim of this study was to determine structural and functional integrity of BBB in a mouse model of Sanfilippo type B at different stages of disease. The primary focus was analyzing BBB competence in various brain structures known to experience neuropathological changes.

## Results

### Microvascular ultrastructure in various brain structures of *Naglu* mutant mice

**Figure 1:**
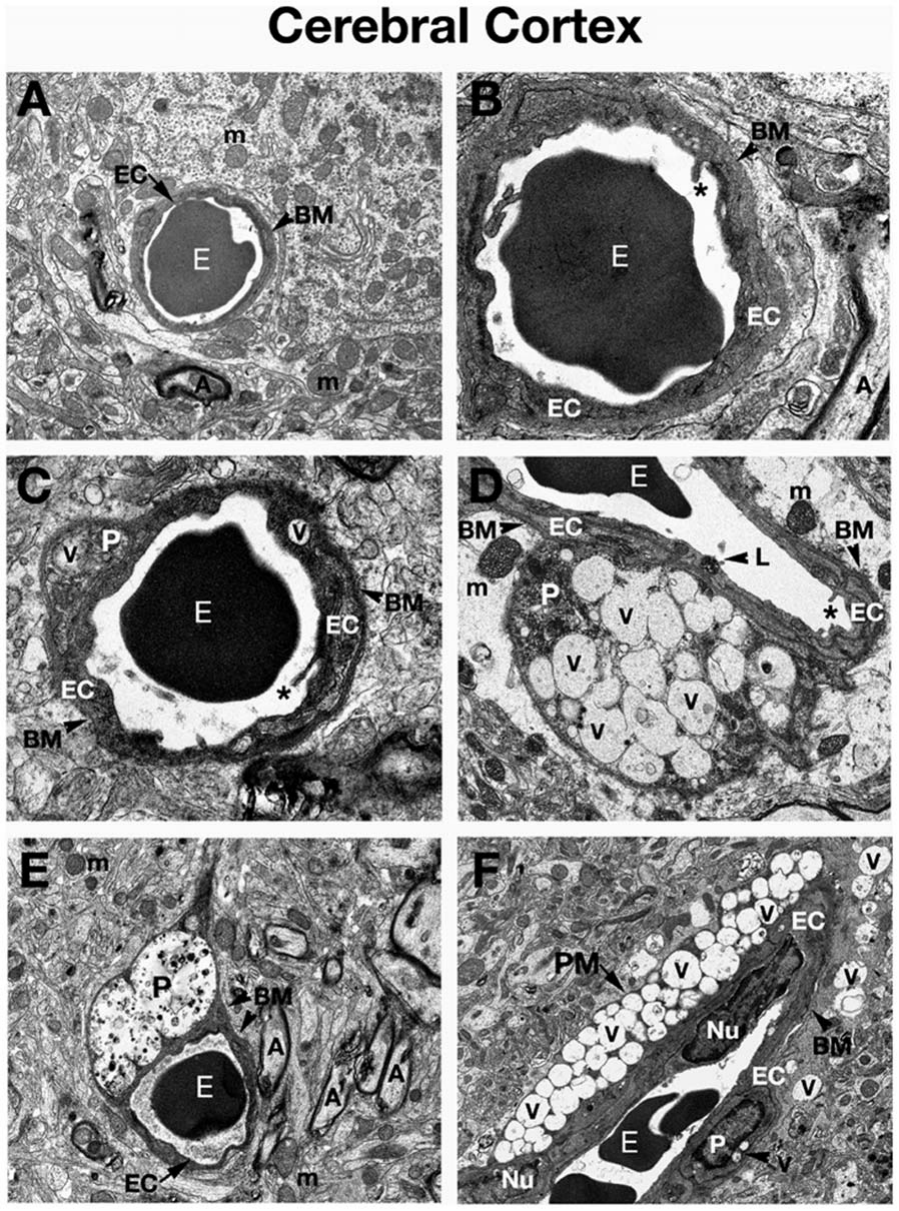
Electron microscope examination of the cerebral cortex of *Naglu* mutant mice. (**A**) Representative area of C57 BL/6J control mouse cerebral cortex characterized by normal ultrastructural appearance of neurons, myelinated axons, and capillaries. (**B**) Capillary at high magnification from the cerebral cortex of control mouse consists of a single layer of endothelial cells surrounded by a layer of basement membrane, forming an intact BBB. Erythrocytes can be observed in the lumen of the capillary. Organelles in all cells were well preserved. (**C**) In the cortex of *Naglu* mutant mouse at 3 months of age (early symptomatic), endothelial cells and pericyte show formation of numerous large vacuoles in their cytoplasm. Microvilli and their fragments can be observed floating free in the capillary lumen. (**D**) Highly vacuolated pericyte was found in contact with microvessel basement membrane in 3 months old mutant mice. Edematous space is observed around vessels. (**E**) In late symptomatic (6 months of age) mutant mouse, large vacuoles containing light and dark material cause swelling of a pericyte, displacing organelles. (**F**) Longitudinal section of capillary showing large perivascular macrophage with numerous vacuoles in its cytoplasm near the blood vessel in the cortex of a 6 month old *Naglu* mouse. Numerous vacuoles were found in cells surrounding the capillary. **EC** - endothelial cell, **BM** - basement membrane, **E** – erythrocyte, **m** – mitochondrion, **A** – axon, **P** – pericyte, **PM** – perivascular macrophage, **Nu** – nucleus, **V** – vacuole, **L** – lysosome, **asterisks** - microvilli. Magnifications: (**A**), (**D**), (**F**): 7,100x; (**B**), (**C**): 14,000x; (**E**): 4,400x.

Brain structures of control mice (10–12 months old) were characterized by normal appearance of neurons, myelinated axons, capillaries, and surrounding astrocytes (Figures 1–4, A, B). All blood vessels were surrounded by healthy neuroglia cell processes. Erythrocytes were observed in the lumen of the capillaries. Cell organelles were well preserved and mitochondria showed a normal pattern of cristae. Capillaries consisted of a single layer of endothelial cells surrounded by a layer of basement membrane, forming an intact BBB.

**Figure 2 pone-0016601-g002:**
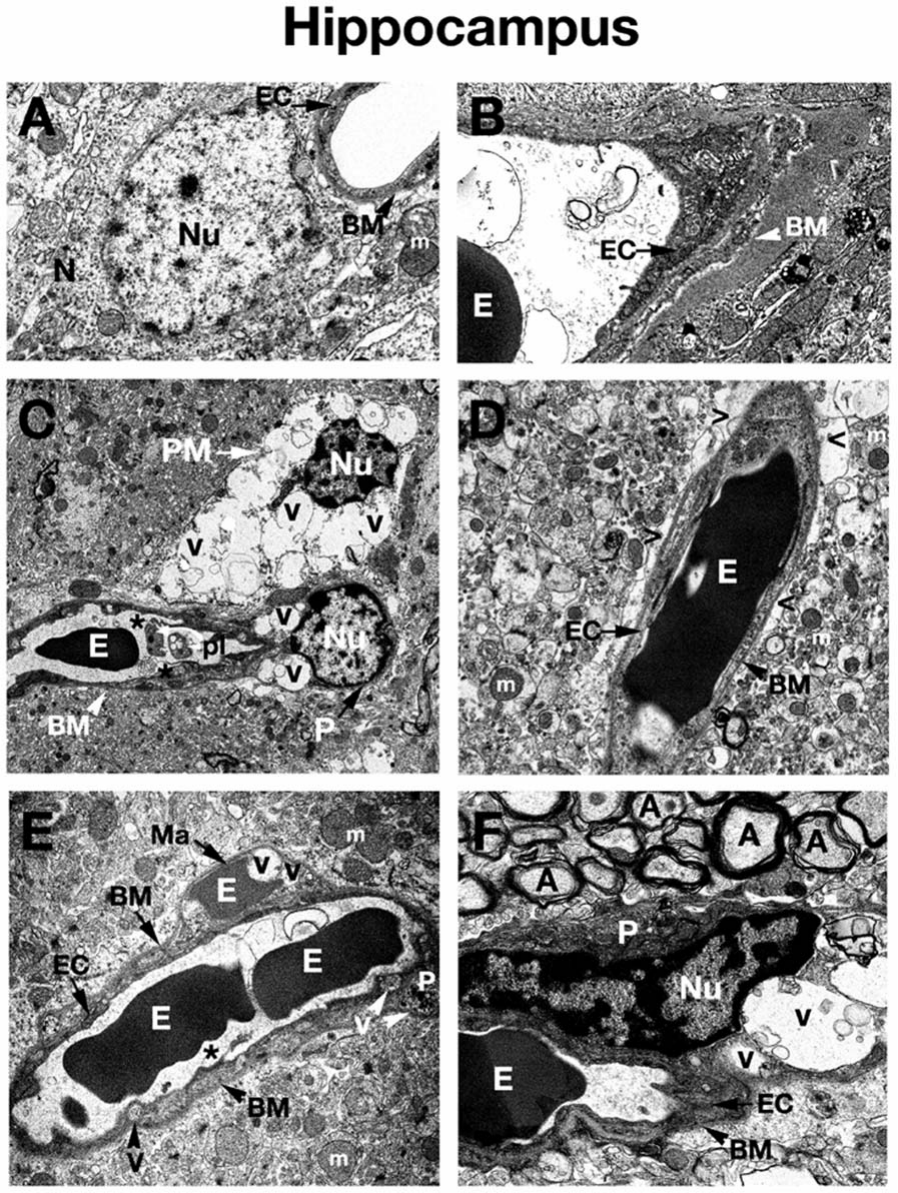
Electron microscope examination of the hippocampus of *Naglu* mutant mice. (**A**), (**B**) Hippocampal area of C57 BL/6J control mouse shows typical normal ultrastructure of neurons and all capillary components. (**C**) Vacuolated endothelial cells and pericytes can be observed in 3 months old *Naglu* mouse. A highly vacuolated perivascular macrophage was also noted. Platelets attracted to a region of endothelial cell damage are visible. (**D**) Perivascular space can be observed around the capillaries in the hippocampus of an early symptomatic mouse at 3 months of age. (**E**) In *Naglu* mouse at 6 months of age, a microaneurysm is visible in the hippocampus, adjacent to a ruptured endothelium. In the microaneurysm, an erythrocyte can be seen trapped under the basement membrane, portending imminent aneurysm rupture. (**F**) Under high magnification, distended nucleus of pericyte with large vacuoles can be observed in late symptomatic mutant mouse. **EC** - endothelial cell, **BM** - basement membrane, **E** – erythrocyte, **m** – mitochondrion, **A** – axon, **N** – neuron, **P** – pericyte, **PM** – perivascular macrophage, **Nu** – nucleus, **V** – vacuole, **Ma** – microaneurysm, **pl** – platelets, **asterisks** - microvilli, **>** - extracellular edematous space. Magnifications: (**A**), (**B**), (**D**), (**E**): 7,100x; (**C**): 3,500x; (**F**): 8,900x.

**Figure 3 pone-0016601-g003:**
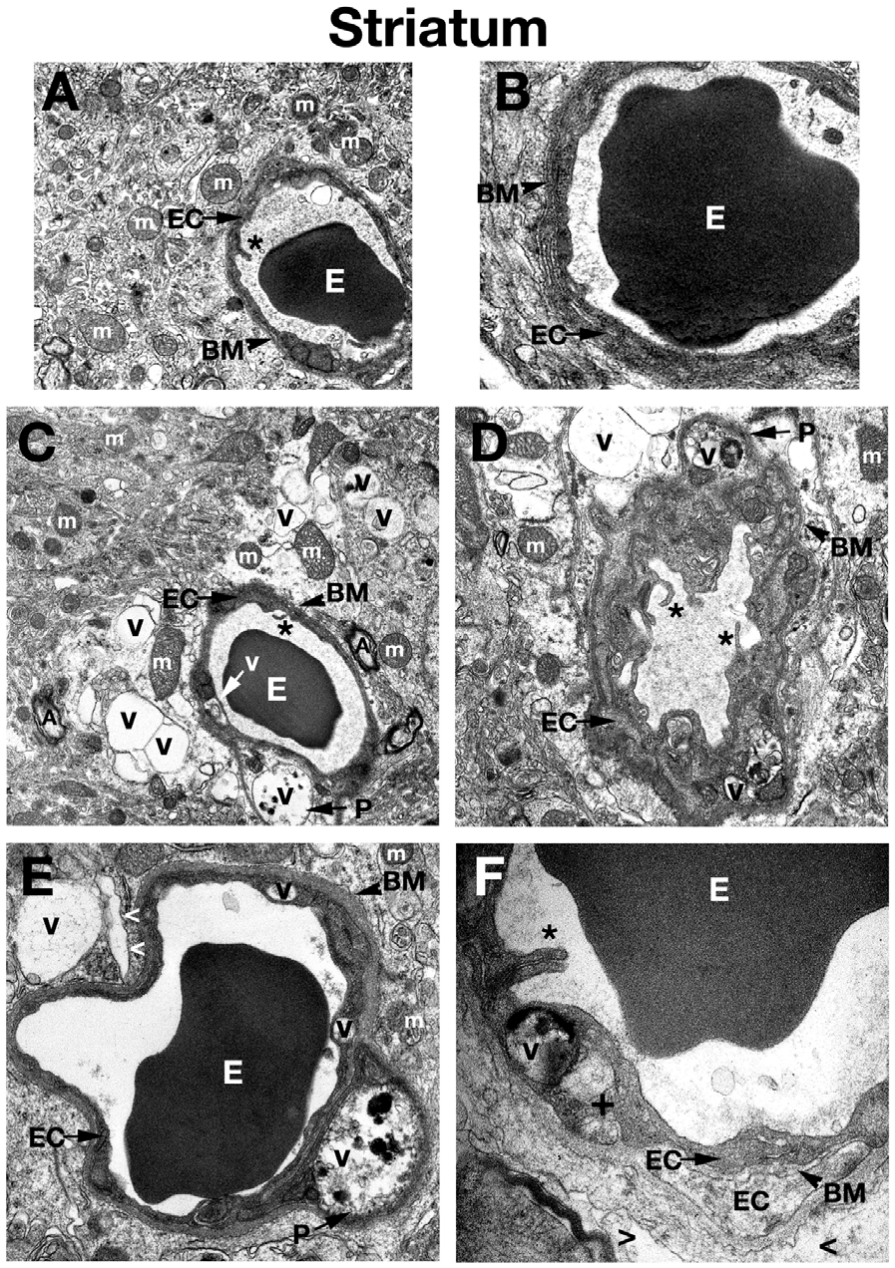
Electron microscope examination of the striatum of *Naglu* mutant mice. (**A**) Structural integrity of capillaries in the striatum was normal in C57 BL/6J control mice. (**B**) A single layer of endothelial cells surrounded by a layer of basement membrane in control mouse seen at high magnification. (**C**), (**D**) Edematous space and large vacuoles are indicated around vessels. Vacuolated pericytes can be seen in the striatum of early symptomatic *Naglu* mice. (**E**) Large vacuoles were found in endothelial cells and pericytes of late symptomatic animals. (**F**) A high magnification image of a capillary from the striatum of a 6 month old mutant mouse showing an endothelial cell with endoplasmic reticulum swelling and formation of a large vacuole in its cytoplasm. Edematous space has appeared around the capillary. Multiple layers of endothelial cells and basement membrane can be observed here, indicating a reparative process taking place. The luminal endothelial cells are damaged**. EC** - endothelial cell, **BM** - basement membrane, **E** – erythrocyte, **m** – mitochondrion, **A** – axon, **P** – pericyte, **PM** – perivascular macrophage, **Nu** – nucleus, **V** – vacuole, **+** - swollen endoplasmic reticulum, **asterisks** - microvilli, **>** - extracellular edematous space. Magnifications: (**A**), (**C**): 7,100x; (**B**), (**E**): 14,000x; (**D**): 8,900x; (**F**): 28,000x.

**Figure 4:**
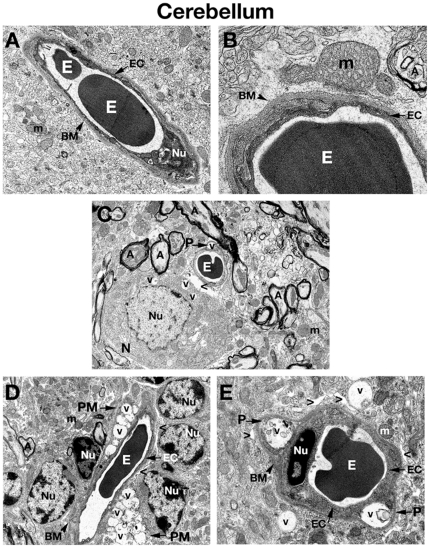
Electron microscope examination of the cerebellum of *Naglu* mutant mice. (**A**), (**B**) Similar to ultrastructure of microvessels in the cerebral cortex, hippocampus, and striatum, the intact BBB in the cerebellum of a C57 BL/6J control mouse consists of an endothelial cell and a single layer of basement membrane. (**C**) Edematous space around vessel as well as vacuolated pericyte is found in the cerebellum of 3 months old mutant mouse. Vacuoles are also seen in the cytoplasm of neuron located near vessel. (**D**) In 6 months old *Naglu* mice, two highly vacuolated perivascular macrophages surround a microvessel. Edematous space around vessels is in contact with astrocytic end-feet. (**E**) Pericytes, which contain large vacuoles displacing and destroying cell organelles, can be seen under the capillary basement membrane in mouse at the same stage of disease. Large vacuoles are also visible outside vessel. **EC** - endothelial cell, **BM** - basement membrane, **E** – erythrocyte, **m** – mitochondrion, **A** – axon, **N** – neuron, **P** – pericyte, **PM** – perivascular macrophage, **Nu** – nucleus, **V** – vacuole, **>** - extracellular edematous space. Magnifications: (**A**) 7,100x; (**C**), (**D**): 4,400x; (**B**): 28,000x; (**E**): 11,000x.

Three months old *Naglu* mice (early symptomatic), displayed ultrastructural abnormalities in capillary endothelia of the cortex (Figure 1, C, D), hippocampus (Figure 2, C, D), striatum (Figure 3, C, D), and cerebellum (Figure 3C). Endothelial cells (ECs) showed endoplasmic reticulum swelling and numerous large vacuoles in cell cytoplasm. Unusually large lysosomes containing dark inclusions were detected in cytoplasm of endothelial cells. (Figure 1D). Blood vessels were surrounded by either degenerating astrocytes or edematous space. Large vacuoles were noted adjacent to capillaries. Neurons with numerous cytoplasmic vacuoles were detected. Microvilli and their fragments were observed floating free in the capillary lumen. Highly vacuolated perivascular macrophages, containing storage products, were found in contact with microvessels in the cortex and hippocampus (Figure 2C). Pericytes surrounding capillaries also contained numerous large vacuoles in their cytoplasm causing cell swelling in all examined brain structures (Figure 1, C, D), (Figure 2C), (Figure 3, C, D), (Figure 4C). Platelets attracted to the region of endothelial cell damage were found in the hippocampus (Figure 2C).

In 6 months old *Naglu* mice (late symptomatic), severe vascular damage was observed in both capillaries and large vessels from all analyzed regions of the brain. In addition to vascular abnormalities described above in 3 months old *Naglu* mice, further swelling and vacuolation of the capillary endothelium was noted. Vacuoles containing light and dark material causing swollen EC and pericytes were found in the cortex (Figure 1E), hippocampus (Figure 2F), striatum (Figure 3E), and cerebellum (Figure 4, D, E). Enlarged pericyte nuclei in the hippocampus were observed (Figure 2F). A cerebral (Figure 4E) capillary was surrounded by two pericytes containing large vacuoles which displaced and destroyed cell organelles. Vacuolated perivascular macrophages were also seen in the cortex (Figure 1F) and cerebellum (Figure 4D). In some microvessels, a single layer of endothelium was observed, while others were surrounded by multiple layers of endothelial cells and basement membrane indicating a reparative process (Figure 3F). Perivascular space could be observed around the capillaries. A microaneurysm was visible in the hippocampus, adjacent to a ruptured endothelium. In the microaneurysm, an erythrocyte could be seen trapped under the basement membrane, portending the aneurysm's imminent rupture (Figure 2E). Interestingly, myelinated axons and dendrites filling the neuropil surrounding capillaries were not destroyed in all examined brain areas of *Naglu* mice at early or late stage of disease. Note, all described ultrastructural BBB abnormalities were similar in both mutant male and females.

### Detection of Evans blue and albumin leakage in various brain structures of *Naglu* mutant mice

**Figure 5:**
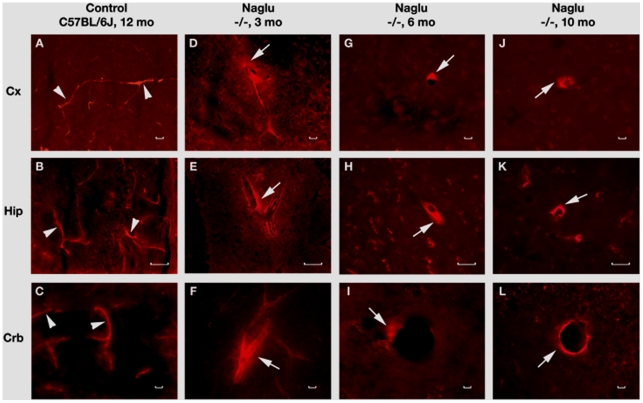
Evans Blue (EB) fluorescence in the brains of *Naglu* mice. In the brain, EB can be clearly detected within the blood vessels (**A**, **B**, **C**, red, arrowheads) in the control C57 BL/6J mouse at 12 months of age. In *Naglu* mice, vascular leakage of EB (red, arrows) is visible in various brain structures (**D**, **E**, **F**) at early (3 months of age), (**J**, **H**, **I**) at late (6 months of age), and (**J**, **K**, **L**) at end stage of disease (10 months of age). Significant EB diffusion into the brain parenchyma from many blood vessels can be detected in end-stage *Naglu* mouse (10–12 months of age). **Cx** – cerebral cortex, **Hip** – hippocampus, **Crb** – cerebellum. Scale bar in A through L is 25 µm.

**Figure 6:**
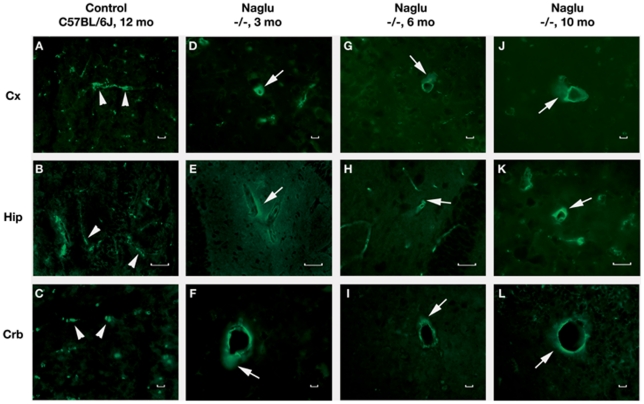
Immunohistochemical staining of albumin in the brains of *Naglu* mice. Albumin immunostaining can clearly be seen within the blood vessels (**A**, **B**, **C**, green, arrowheads) of control C57 BL/6J mouse at 12 months of age. In *Naglu* mice, albumin extravasation (green, arrows) can be seen in various brain structures (**D**, **E**, **F**) at early (3 months of age), (**G**, **H**, **I**) at late (6 months of age), and (**J, K, L**) end stages of disease (10 months of age). **Cx** – cerebral cortex, **Hip** – hippocampus, **Crb** – cerebellum. Scale bar in A through L is 25 µm.

The functional competence of the BBB in *Naglu* mice at different stages of disease was determined by examining vascular leakage of EB, 961 Da, and albumin, 68 kD. In control wild type mice at 3, 6 or 10-12 months of age, EB (Figure 5, A–C) and albumin (Figure 6, A–C) were clearly detected within the blood vessels of the brain. Vascular leakage of EB and albumin was observed in early (3 months of age) (Figures 5 and 6, D–F) and late symptomatic *Naglu* mice (6 months of age) (Figures 5 and 6, G, H, I) in various brain structures. Significant EB and albumin diffusion into the parenchyma of the brain from multiple blood vessels was detected in both male and female end stage *Naglu* mice (10–12 months of age) (Figures 5 and 6, J–L). Although the entire sectioned brain was analyzed for vascular leakage, the majority of blood vessel leakage was detected in the cerebellar lobules (6–9Cb), cerebral cortex (M2), hippocampus (CA1, CA2), and midbrain of mutant mice at all disease stages (Table 1). Although both EB and albumin extravasation abnormalities were commonly found in the same microvessels (Figures 5 and 6, E, K, L), some capillaries showed only EB leakage. Interestingly, neither EB nor albumin leakage was indicated in the olfactory bulb. Thus, vascular leakage of EB and albumin was detected in *Naglu* mice not only at late stage of disease but also concurrent with early disease symptoms.

**Table 1:** Pattern of vascular leakage in the brains of *Naglu* mice at different stages of disease.

*Naglu* mice, age	Cx	Hip	Cb	Md	MB	OB	PS	ST	TH
	EB/Alb	EB/Alb	EB/Alb	EB/Alb	EB/Alb	EB/Alb	EB/Alb	EB/Alb	EB/Alb
**3 mo**	++/++	++/++	++/+	++/+	+/--	--/--	+/--	+/+	+/+
**6 mo**	++/++	+/+	++/+	++/+	+/+	--/--	+/+	+/+	+/+
**10–12 mo**	++/++	+/ +	++/+	++/+	+/+	--/ --	+/+	+/+	+/+

**Key: Cx** – cerebral cortex, **Hip** – hippocampus, **Cb** – cerebellum, **Md** – medulla, **MB** – midbrain, **OB** – olfactory bulb, **PS** – pons, **ST** – striatum, **TH** – thalamus, **EB** – Evans Blue, **Alb** – albumin, **--** - no leak, + - moderate leak (near abluminal side of the capillaries), **++** - significant leak (at a distance from the capillaries). Note: no vascular leakage was determined in examined brain blood vessels from control mice at 3, 6 or 10–12 months of age.

### Immunohistochemical staining for GM3 ganglioside in brains of *Naglu* mice

**Figure 7:**
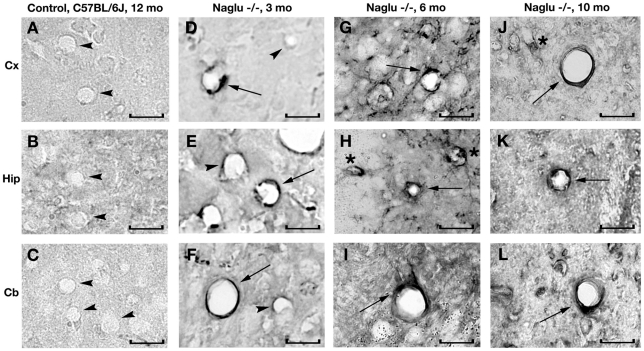
Immunohistochemical staining for GM3 ganglioside in the brains of *Naglu* mice. In the brain of control C57 BL/6J mouse at 12 months of age, GM3 ganglioside is not detected within the vascular endothelium of blood vessels (**A**, **B**, **C**, arrowheads). In early symptomatic (3 month of age) *Naglu* mice, some endothelial cells stained positive for GM3 (**D**, **E**, **F**, arrows) and some cells were negative for GM3 (**D**, **E**, **F**, arrowheads). GM3 accumulation (arrows) was seen in the endothelia of numerous brain blood vessels (**G**, **H**, **I**) at late (6 months of age) and (**J**, **K**, **L**) at end stages of disease (10 months of age). Significant GM3 ganglioside accumulation was also seen in neurons (asterisks) in *Naglu* mice. **Cx** – cerebral cortex, **Hip** – hippocampus, **Crb** – cerebellum. Scale bar in A through I is 25 µm.

Significant GM3 ganglioside accumulation was seen in neurons and glial cells in both male and female *Naglu* mice. GM3 ganglioside was not detected within the brain endothelium of blood vessels (Figure 7, A–C) in control wild type mice. In early symptomatic (3 months old) *Naglu* mice, some endothelial cells stained positive for GM3 (Figure 7, D–F). More GM3 accumulation was seen in the endothelia of numerous brain blood vessels at late (6 months of age) and end stages of disease (10 months of age) in the cerebral cortex (Figure 7, G, J), hippocampus (Figure 7, H, K), and cerebellum (Figure 7, I, L).

## Discussion

In the present study, we investigated the structural and functional integrity of the blood-brain barrier in a mouse model of Sanfilippo type B (MPS III B) at different stages of disease. The primary focus was determining BBB competence in brain structures known to experience neuropathological changes such as the cerebral cortex, hippocampus, striatum, and cerebellum. Major findings of our study were: (1) capillary ultrastructure revealed endothelial cell damage and astrocyte degeneration which compromised the BBB, resulting in vascular leakage, (2) endothelial cells showed endoplasmic reticulum swelling and formation of numerous large cytoplasmic vacuoles, degeneration of cytoplasmic organelles, abnormal microvilli formation, and shedding of fragments of cell membrane and microvilli, (3) edematous space was detected surrounding microvessels, (4) pericyte degeneration was observed in the large majority of damaged vessels, (5) highly vacuolated perivascular macrophages were found in contact with microvessels, (6) a microaneurysm was visible adjacent to a ruptured endothelium, (7) EB and albumin microvascular leakage was clearly indicated in multiple brain structures, (8) GM3 ganglioside accumulation was determined in brain microvasculature endothelium. Structural and functional impairment of the BBB in the MPS III B mouse model is a novel finding. This new discovery of microvascular damage in mice even at early disease stage may have implications for disease pathogenesis.

The BBB structure and function have been detailed in numerous reviews [13]–[15], [21] emphasizing the BBB's neuroprotective role in maintaining a stable fluid environment in the brain. This unique microvascular barrier in the brain prevents the passive diffusion of many harmful solutes from blood. Specific transport systems allow influx of various required substances and efflux of cell products to maintain CNS homeostasis [21], [22]. However, BBB dysfunction or damage to any structural BBB elements would likely unbalance the fragile CNS equilibrium. Although compromised BBB integrity has been shown in some lysosomal storage diseases (reviewed in [16]), this is the first report demonstrating microvascular endothelial cell damage in brains of mice modeling MPS III B. The numerous vacuoles detected in endothelial cell cytoplasm, even in early symptomatic mice, may degrade cell function leading to BBB impairment. This barrier damage was confirmed by vascular leakage of Evans Blue and albumin resulting from endothelium damage in MPS III B mice, similar to extravasation of Evans Blue and endogenous IgG findings in mouse models of Sandhoff disease and GM1 gangliosidosis [17]. Jeyakumar et al. [17] also revealed that edematous endothelial cells with large vacuoles including membranous cytoplasmic bodies were indicated in ultrastructure of some brain areas of these mice. Capillary endothelial cell dysfunction, potentially allowing harmful blood-borne solutes including neurotoxins to enter the CNS, may accelerate neuropathological changes in MPS III B. Alternatively, damaged endothelial cells may alter specific mechanisms for transport of various solutes across the BBB. In this scenario, CNS neural cells might suffer the dual effects of reduced nutrition and increased metabolite levels, impairing CNS function.

Additional to endothelial cell damage, pericyte degeneration was observed in the majority of capillaries from examined brain structures in early and late symptomatic MPS III B mice. Large vacuoles displacing and destroying cell organelles were seen in most pericytes surrounding capillaries. Pericytes are believed to be cells of mesodermal origin derived from mesenchymal precursor cells and during angiogenesis remain enclosed within the basal lamina. A number of reviews have revealed the important role of pericytes in maintaining BBB integrity [23]–[26]. Due to their location and properties, pericytes have been proposed as contractile cells regulating blood flow and vascular tone. Imperative pericyte function includes vessel permeability regulation, control of microvascular remodeling and new capillary growth, immunologic defense, matrix protein synthesis, and interaction with endothelial and other cells providing vessel stabilization. Interactions between pericytes and endothelial cells, via secreted soluble factors such as platelet-derived growth factor and transforming growth factor beta 1, are vital to the growth and maintenance of vessels [24], [27]. Thus, damage to both capillary endothelial cells and pericytes in MPS III B mice as determined via electron microscopy might severely compromise BBB.

Another of our findings was that perivascular macrophages in close contact with capillary basement membrane were highly vacuolated. Large vacuoles fully covered the cell cytoplasm similar to microglial cells with great inclusions [12]. However, perivascular macrophages are cells with many properties distinct from pericytes, microglia, and macrophages even though sharing similar morphological appearance as blood-derived macrophages [28] and importantly maintain Òperivascular spaces as drainage pathways for soluble and insoluble material from the brainÓ [29]. Also, perivascular macrophages play an essential role during CNS inflammation by facilitating the influx of leukocytes through the BBB [30]. Future studies are needed to investigate inflammatory processes in relation to BBB alterations in MPS III B mice.

It is our belief that the primary insult to BBB integrity is likely from accumulated storage products within cellular components of the BBB. Our data show accumulation of GM3 ganglioside, a secondary storage product, not only in neurons and glial cells at different stages of disease, but also in brain endothelium of MPS III B mice at early disease stage, making it the most likely effector damaging endothelial cells. Importantly, more GM3 accumulation was seen in the endothelia of numerous brain blood vessels as disease progressed.

Thus, endothelial and perivascular (pericytes and perivascular macrophages) cell damage, detected in a majority of blood vessels in different brain structures of MPS III B mice at early (3 months of age) and late (6 months of age) stages of disease, compromised BBB integrity and led to vascular leakage. However, it is possible that BBB dysfunction occurred prior to or concurrent with the appearance of neuropathological changes (1 month-old *Naglu* mice). Also, observed differences in the degree of vascular leakage for EB and albumin from different brain structures of MPS III B mice will lead us to investigate functional vascular pathology in younger mice. Surprisingly, more capillary leakage was seen in some brain structures (i.e. hippocampus) of early symptomatic mice (3 months of age) or similar (i.e. cerebral cortex [M2] and cerebellar lobules [6–9Cb]) than late symptomatic animals (6 and 10–12 months of age) (see Table 1). Currently, no definitive explanation can be made for this differential leakiness. One of our speculations is that the observed brain regions differ in metabolic functional activity, especially in growing animals, and higher activity may require more substantial exchanges of nutrition and metabolites. If the BBB is already weakened in these brain areas, more vascular leakage might occur. Quantitative analysis of dye extravasation will be necessary to further investigate differential leakage and is planned for our future studies. Collectively, determining the evolution of BBB dysfunction in MPS III B is important not only for understanding this additional aspect of disease pathogenesis, but is also critical to the development of a successful MPS IIIB therapy. Since our study is ongoing, some clarification regarding possible mechanism(s) of BBB impairment is expected.

Special attention should be given to the possibilities for cerebral hemorrhages in MPS III B. A microaneurysm adjacent to a ruptured endothelium in the hippocampal area was detected in a late symptomatic mouse. Intracerebral hemorrhage is a serious condition typically resulting from damage in the bleeding brain area and is a common cause of stroke. This hemorrhage may have been caused by weakened BBB competence, especially of some structural components. Although this is the first microaneurysm reported in MPS III B mice, awareness of the possibility for microaneurysms in human MPS III B patients is important.

Together, our study results clearly demonstrate BBB impairment in various brain structures in a mouse model of MPS III B at different stages of disease. It is important to confirm this BBB impairment as an additional mechanism of disease pathogenesis in MPS III patients, an investigation which will be the focus of our future study. The likelihood of severely damaged endothelial and perivascular cells leading to breakdown of BBB integrity should be considered in treatment development for MPS III B, particularly in regards to drug delivery across the blood-brain barrier. Restoration of BBB integrity is essential and should benefit patients in numerous ways including re-establishing neuroprotection.

## Materials and Methods

### Animals

All described procedures were approved by the Institutional Animal Care and Use Committee at USF and conducted in compliance with the *Guide for the Care and Use of Laboratory Animals*. All animals used in the study were obtained from our established colony of *Naglu* mice developed from heterozygous breeding pairs (The Jackson Laboratory, Bar Harbor, Maine). Further development of the knockout strain continued on the background strain C57BL/6J. Heterozygous breeding pairs were used to produce littermates of three phenotypes: homozygote, heterozygote, and wild-type. Genotyping of animals was performed by B6.129S6-*Naglu^tm1Efn^* probe hybridization assay via manufacture's protocol (Transnetyx, Inc., Cordova, TN) of tail biopsy (10 mg of tissue) at 10–15 days of age. Thirty three mice were used: 8 early symptomatic (4 males, 4 females, 3 months of age, homozygotes), 8 late symptomatic (4 males, 4 females, 6 months of age, homozygotes), 4 end stage (2 males, 2 females, 10–12 months of age, homozygotes), and 13 wild type C57BL/6J mice of the same ages (1 male, 1 female, 3 months of age; 2 males, 1 female, 6 months of age; 5 males, 3 females, 10–12 months of age). Microvessel ultrastructure in various brain regions (cerebral cortex, hippocampus, cerebellum, and striatum) from mutant mice at early or late stage disease and wild type mice was examined with an electron microscope (EM). Functional integrity of the BBB was determined by Evans blue dye and albumin leakage via immunohistochemistry.

### Evans Blue dye

Evans Blue dye (EB, Aldrich Chemical), 961 Da, was used as a tracer for assessing BBB disruption. *Naglu* (n = 2 of each sex per group: 3, 6, 10–12 months of age) and wild type (n = 2 of each sex, 10–12 months of age) mice were intravenously injected with 2% EB in saline solution via the jugular vein 30–40 min prior to euthanasia. The surgical procedure was performed as we previously described [19]. Briefly, mice were anesthetized with Isoflurane, delivered using a calibrated vaporizer equipped induction chamber and nose cone and administered at 2–5% in O2 (2 L/min) to induce anesthesia and then decreased to 2% to maintain anesthesia. The jugular vein was exposed and isolated using blunt dissection. The vein was ligated and a 31-gauge needle, attached to a 100-µl Hamilton syringe was placed into the lumen of the vein and sutured in place. Evans Blue was delivered (0.2 ml/100g, 40 µl/mouse) during 2 min. The needle was withdrawn, the suture tightened, and the incision closed with Vetbond.

### Euthanasia and tissue preparation

Euthanasia of all mice was achieved under deep pentobarbital (150 mg/kg) anesthesia. Perfusion was not performed, avoiding any mechanical disruption or collapse of blood capillaries and allowing determination of BBB condition without external manipulations for delivery washing/fixative solutions similarly to our previously published studies [19], [20]. Additionally, using non-perfused mice allowed observations of administered EB dye and albumin *within* or *outside* blood vessels depending upon BBB condition. Mice receiving an EB injection were euthanized 30–40 min after injection. Brains were immediately removed, fixed intact in 4% paraformaldehyde (PFA) in 0.1 M phosphate buffer (PB), pH 7.2, for 24 hrs and then cryoprotected in 20% sucrose in 0.1 M PB (pH 7.2) overnight. Sagittal brain sections were cut at 30 µm in a cryostat and used for immunohistochemical analysis of EB leakage. Mice assayed for EM analysis were also euthanized as described above. Their brains were immediately removed and fixed in 4% PFA in 0.1 M PB, pH 7.2, for 16–24 hours at 4°C. The next day, brains were cut into 1 mm slices, mapped against a diagram of the whole slice, and regions to be studied were removed from the slices and fixed overnight in 2.5% glutaraldehyde in 0.1 M PB (Electron Microscopy Sciences, Inc., Hatfield, PA) at 4°C. The following day, tissues were transferred to a fresh change of the above buffer and stored for further EM processing.

### Electron microscopy

The BBB structural characteristics were identified in different brain structures of *Naglu* mutant male and female mice at 3 and 6 months of age and compared to wild type control mice (10–12 months old) using electron microscopy. Structural integrity of microvessels [20] was analyzed in the cortex (M2), hippocampus (dentate gyrus), striatum (caudate putamen), and cerebellum (6–9 Cb) of the brain. Briefly, tissue samples were post-fixed in 1% osmium tetroxide (Electron Microscopy Sciences, Inc., Hatfield, PA) in 0.1 M PB for 1 hour at room temperature (RT). Following osmication, the tissues were dehydrated at RT in a graded series of acetone dilutions (30%, 50%, 70%, and 95% acetone in water), 15 minutes each change. Three 15-minute changes in 100% acetone were then made and the tissues were transferred to a 50∶50 mix of acetone and LX112 epoxy resin embedding mix (Ladd Research Industries, Burlington VT). The tissues were infiltrated with this mix for 1 hour under vacuum. The tissues were then transferred to a 100% LX112 embedding mix and infiltrated for 1 hour on a rotator. Two more 1-hour infiltration steps were performed with fresh changes of the embedding mix. The tissues were further infiltrated overnight in fresh embedding medium at 4°C. On the following day, the tissues were infiltrated in two additional changes of embedding medium at RT, 4 hours per change, and then embedded in a fresh change of resin in tissue capsules. The blocks were polymerized at 70°C in an oven overnight. The blocks were trimmed and then sectioned with a diamond knife on an LKB Huxley ultramicrotome. Thick sections cut at 0.35 µm were placed on glass slides and stained with 1% toluidine blue stain. Thin sections were cut at 80–90 nm, placed on copper grids, and stained with uranyl acetate and lead citrate.

### Detection of BBB condition

For analysis of BBB ultrastructure, brain structures (cerebral cortex, hippocampus, cerebellum, and striatum) were examined and photographed with a FEI Morgagni transmission electron microscope (FEI, Inc., Hillsboro, OR), using an Olympus MegaView III digital camera (ResAlta Research Technologies Corp., Golden, CO.) at 60 kV. For identification of EB, serial tissue sections of the brain were thaw-mounted on slides, washed with deionized water to remove the freezing medium, and then rinsed several times in phosphate-buffered saline (PBS). The slides were coverslipped with Vectashield (Vector Laboratories) and examined under an Olympus BX60 epifluorescence microscope by an assistant blinded to mouse genotype and age.

### Immunohistochemistry

Immunohistochemical staining for mouse albumin was performed to determine vascular integrity in the brain. Brain tissues were blocked for 60 min prior to applying goat anti-mouse serum albumin antibody conjugated to FITC (1∶300, Alpha Diagnostic International). Tissue sections were then incubated for 2 hrs at RT. The brain tissues were rinsed three times in PBS and then coverslipped with Vectashield (Vector Laboratories). Immunohistochemical staining for GM3 ganglioside was performed to detect this secondary storage product in the brains of *Naglu* mice as previously described [10]. Serial tissue sections of mutant and control mouse brains were rinsed several times in PBS to remove the freezing medium. The brain tissues were then incubated in a blocking solution of 10% normal goat serum (NGS) and 0.3% Triton X-100 in PBS for 60 min at RT. Mouse monoclonal antibody GM3 (1∶100, NeuAc, Cosmo Bio Co., LTD, Tokyo, Japan) with 1.5% NGS, and 0.3% Triton X-100 in PBS was applied on the slides overnight at 4°C. The slides were rinsed in PBS and incubated with biotin-labeled goat-anti-mouse secondary antibody (1∶200, Vector) for 60 min at RT followed by an avidin-biotin-peroxidase (ABC-Elite kit, Vector) with 3,3-diaminobenzidine (DAB, Vector). The sections were examined using an Olympus BX60 microscope.
